# Development of ^89^Zr-Ontuxizumab for *in vivo* TEM-1/endosialin PET applications

**DOI:** 10.18632/oncotarget.7552

**Published:** 2016-02-21

**Authors:** Sara E.S. Lange, Alex Zheleznyak, Matthew Studer, Daniel J. O'Shannessy, Suzanne E. Lapi, Brian A. Van Tine

**Affiliations:** ^1^ Division of Medical Oncology, Washington University in St. Louis, St. Louis, MO, USA; ^2^ Department of Radiology, Washington University in St. Louis, St. Louis, MO, USA; ^3^ Translational Medicine and Diagnostics, Morphotek Inc., Exton, PA, USA; ^4^ Siteman Cancer Center, Washington University in St. Louis, St. Louis, MO, USA

**Keywords:** ^89^Zr, Ontuxizumab, sarcoma, TEM-1, immuno-PET

## Abstract

**Purpose:**

The complexity of sarcoma has led to the need for patient selection via *in vivo* biomarkers. Tumor endothelial marker-1 (TEM-1) is a cell surface marker expressed by the tumor microenvironment. Currently MORAb-004 (Ontuxizumab), an anti-TEM-1 humanized monoclonal antibody, is in sarcoma clinical trials. Development of positron emission tomography (PET) for *in vivo* TEM-1 expression may allow for stratification of patients, potentially enhancing clinical outcomes seen with Ontuxizumab.

**Results:**

Characterization of cell lines revealed clear differences in TEM-1 expression. One high expressing (RD-ES) and one low expressing (LUPI) cell line were xenografted, and mice were injected with ^89^Zr-Ontuxizumab. PET imaging post-injection revealed that TEM-1 was highly expressed and readily detectable *in vivo* only in RD-ES. *In vivo* biodistribution studies confirmed high radiopharmaceutical uptake in tumor relative to normal organs.

**Experimental Design:**

Sarcoma cell lines were characterized for TEM-1 expression. Ontuxizumab was labeled with ^89^Zr and evaluated for immunoreactivity preservation. ^89^Zr-Ontuxizumab was injected into mice with high or null expressing TEM-1 xenografts. *In vivo* PET imaging experiments were performed.

**Conclusion:**

^89^Zr-Ontuxizumab can be used *in vivo* to determine high versus low TEM-1 expression. Reliable PET imaging of TEM-1 in sarcoma patients may allow for identification of patients that will attain the greatest benefit from anti-TEM-1 therapy.

## INTRODUCTION

The utility of biomarkers in the diagnosis, staging, and determination of clinical response to treatment is emerging as an invaluable tool in the care of cancer patients [1-4]. The use of a biomarker to not only image a malignancy, but to offer guidance as to which therapy would most benefit a patient is at the core of personalized therapy. The ability to delineate a patient's clinical response to a biologic treatment would allow for patient stratification into groups that would either benefit from a treatment, or spare patients the costs and potential toxicities of ineffective treatments [5-7].

Tumor endothelial marker-1 (TEM-1), also referred to as endosialin or CD248, is a tumor vascular marker that is a 175k-Da type I transmembrane protein of the C-type lectin-like receptor family [5, 8-10]. It is most closely related to the family of transmembrane glycoproteins that includes thrombomodulin, functions in the angiogenesis process, and may also be involved in cell adhesion and migration. Its role in other cellular functions is not fully defined [6, 11-13]. TEM-1 is primarily expressed on tumor stroma and tumor vessels in multiple human cancers, but is not expressed in normal adult tissue or blood vessels [9, 10, 14-16]. A limited number of tumors have also been demonstrated to express TEM-1. By immunohistochemistry (IHC), the expression of TEM-1 is restricted to stromal cells and tumor-associated perivascular cells in ovarian, breast, and lung cancers [17, 18]. In sarcoma, the expression of endosialin/CD248/TEM-1 has been examined in cell culture, clinical specimens and animal models, and endosialin was found to be expressed frequently and at high levels in perivascular cells, stromal cells. The malignant cells themselves [17, 19, 20]. A high level of endosialin expression on primary clinical samples of sarcoma suggests the potential utility of targeting endosialin/CD248/TEM-1 on sarcomas *in vivo* [17, 19, 20].

Previous work using a ^124^I-labled anti-TEM-1 antibody illustrated the feasibility of using PET imaging to visualize this target in a cell line engineered to express high levels of TEM-1 [5]. Using ^125^I-labled Ontuxizumab TEM-1 positive tumor bearing mice were found to clear the antibody at a much slower rate that TEM-1 negative tumor bearing mice [5]. In addition, biodistribution studies demonstrated high uptake of iodinated Ontuxizumab in tumors compared to normal tissues [5]. The aims of the current study were twofold. First, to develop a ^89^Zr-PET imaging agent for imaging of TEM-1 expression as ^89^Zr is known to be a residualizing radionuclide (trapped inside cells after metabolism) and thus has the potential of generating higher quality images [21]. Secondly, we sought to examine the feasibility of imaging TEM-1 expression on non-engineered sarcoma cell lines, *in vivo*, to assess the potential clinical utility of this agent.

At present, the ability to determine the clinical expression of TEM-1 in patients with metastatic disease relies on IHC of biopsies of single lesions or primary tumors that may not represent true TEM-1 expression when compared to the total tumor burden in a patient. Negative staining for TEM-1 may represent an artifact of processing or biopsy selection, while positive staining may only relate to the specific tumor that was biopsied and not the entire tumor burden. We proposed to develop a PET clinical screening test to determine which patients would most likely benefit from anti-TEM-1 therapy using ^89^Zr-Ontuxizumab.

## RESULTS

### TEM-1 expression in sarcoma cell lines

**Figure 1:**
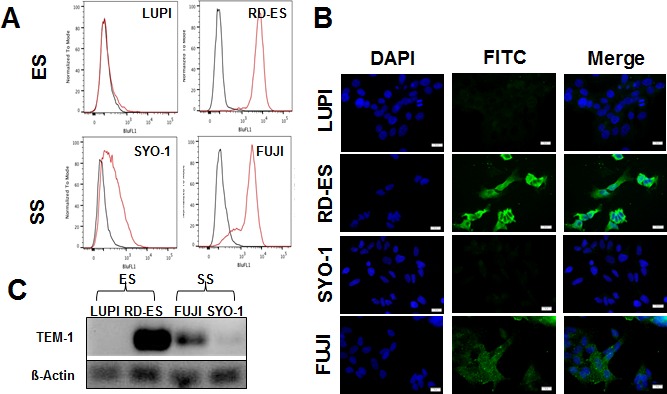
TEM-1 Expression: High and Null Expressing Sarcoma Lines **A.** FACS analysis using Ontuxizumab antibody and FITC-labeled anti-human secondary antibody to determine TEM1 expression. RD-ES (Ewing's sarcoma) and FUJI (synovial sarcoma) show high TEM-1 expression. LUPI, a Ewing's sarcoma line, and SYO-1, a synovial sarcoma line, display null to low expression of TEM-1, respectively. **B.** Semi-quantitative immunofluorescence of TEM-1 expression. Cell lines RD-ES and FUJI display strong Ontuxizumab binding while LUPI and SYO-1 display null binding. Images were captured using an exposure time of 25 msec through a 60x objective keeping time constant across all images. **C.** NATIVE-PAGE Western blot showing high-expressing TEM-1 lines RD-ES and FUJI, with null-expressing lines LUPI and SYO-1.

To identify potential in vivo xenograft candidates, a panel of thirteen sarcoma cell lines was screened for TEM-1 expression by FACS. Three Ewing's cell lines (SK-ES, RD-ES, and LUPI), three osteosarcoma cell lines (MG-63, MNNG-HOS, U2-OS) two synovial sarcoma cell lines (SYO-1, FUJI), two uterine sarcoma cell lines (SK-LMS-1, SK-UT), one fibrosarcoma (HT-1080), one rhabdomyosarcoma (RD), and one malignant peripheral nerve sheath tumor (MPNST) (ST-88-14) were assessed. Indirect FACS using Ontuxizumab and FITC-labeled anti-human secondary antibody revealed that cell lines RD-ES and FUJI displayed very high expression of TEM-1 when compared to background (Figure 1A). Conversely, LUPI and SYO-1 were null and low expressers of TEM-1, respectively (Figure 1A). Binding of Ontuxizumab to these two paired cell lines was confirmed by semi-quantitative immunofluorescence; using fixed camera settings, the FITC-labeled anti-human antibody labeling of bound Ontuxizumab was noted to be positive in RD-ES and FUJI when compared to LUPI and SYO-1 (Figure 1B). Finally we performed NATIVE-PAGE Western blot analysis to confirm the expression of TEM-1 in RD-ES and FUJI when compared to LUPI and SYO-1 (Figure 1C); a rat monoclonal antibody to TEM-1 was used as Ontuxizumab only recognizes a three dimensional structure of TEM-1 that is lost upon immunoblotting. FACS analysis (Supplemental Figure 1) and immunofluorescence (Supplemental Figure 2) of the remaining nine sarcoma cell lines revealed that both SK-ES, a Ewing's sarcoma, and ST-88-14, a MPNST, displayed low to moderate TEM-1 expression, while the remainder of the panel were null expressers of TEM-1.

### DFO conjugation and ^89^Zr labeling does not affect binding or uptake of ontuxizumab

**Figure 2:**
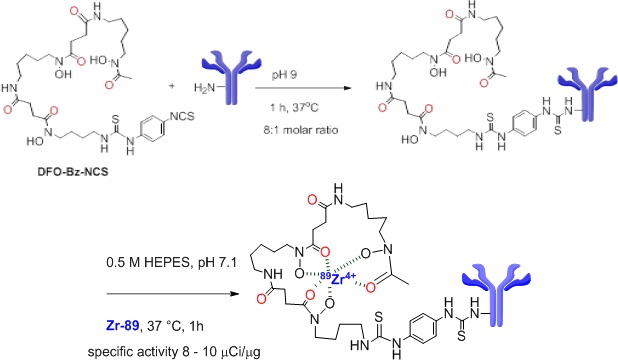
Ontuxizumab conjugation Ontuxizumab was conjugated to *p*-isothiocyanatobenzyl-desferrioxamine (DFO-Bz-NCS metal chelate) *via* lysine residues. After purification, the antibody conjugate was radiolabeled with ^89^Zr-oxalate under neutral pH conditions.

**Figure 3:**
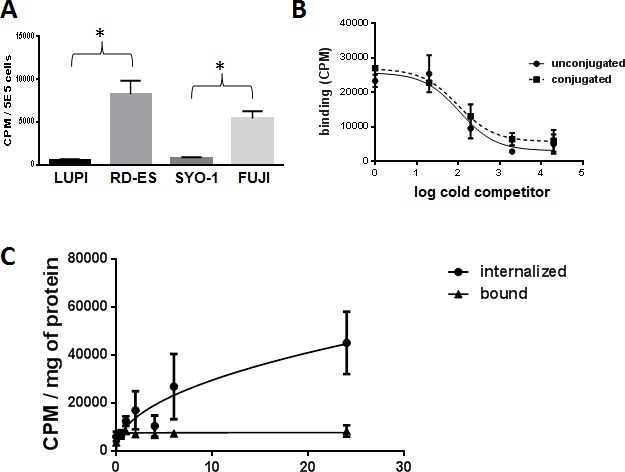
Binding Study and Uptake **A.** ^89^Zr-Df-Bz-NCS-Ab binding to selected sarcoma cell lines. Radiolabeled antibody was allowed to bind to selected cell lines for 35 min as described in the *Methods* section. RD-ES and FUJI cells demonstrated good binding of ^89^Zr-Df-Bz-NCS-Ab, while SYO-1 and LUPI showed no appreciable binding. **B.** Immunoreactivity of ^89^Zr-Df-Bz-NCS-Ab determined using ^89^Zr-Df-Bz-NCS-Ab binding to immobilized TEM-1 in the presence of increasing amounts of Df-Bz-NCS-Ab (conjugated) or Ab (unconjugated). Df-Bz-NCS-Ab IC_50_ = 13.1 nM, Ab IC_50_ = 14.5 nM. Assay performed as described in the *Methods* section.

Ontuxizumab underwent chelator conjugation with ^89^Zr (Figure 2). To determine if ^89^Zr labeling of Ontuxizumab altered binding affinity, we performed binding and uptake assays. Cells were incubated with the radiolabeled antibody, lysed, and cell associated activity detected with a gamma-counter. Protein amount was measured with a BCA assay and the data expressed as counts per milligram of protein (CPM/mg protein). Binding of the radiolabeled antibody was significantly higher for RD-ES and FUJI when compared with LUPI and SYO-1 (Figure 3A). Immunoreactivity of the radiolabeled antibody was determined using ^89^Zr-Df-Bz-NCS-Ab binding to immobilized TEM-1 in the presence of increasing amounts of Df-Bz-NCS-Ab (conjugated) or Ab (unconjugated). When assessing the binding affinity of unconjugated compared to conjugated antibody, there was no significant difference in CPM between conjugated and unconjugated antibody *in vitro*. Therefore, conjugation of the DFO and radiolabeling it with ^89^Zr did not change the antibody's binding properties (Figure 3B).

Another recently reported function of Ontuxizumab is internalization after binding to endosialin [28]. To determine if ^89^Zr altered the ability of Ontuxizumab to internalize after labeling we performed an *in vitro* uptake assay using the TEM-1 expressing cell line RD-ES (Figure 3C). We found that the labeled antibody was internalized slowly, likely at the rate of receptor turnover, and that this was not saturable over 24 hours, thus suggesting that radioimmunotherapy or antibody conjugates using Ontuxizumab may be useful for drug development [29].

### *In vivo* PET imaging and biodistribution

**Figure 4:**
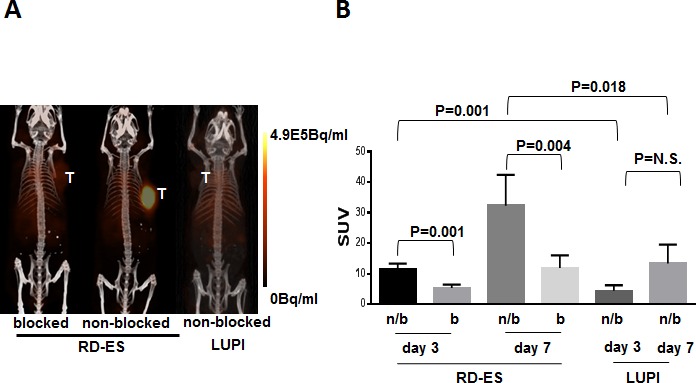
*In vivo* PET Imaging / Radiopharmaceutical tumor uptake **A.** Maximum intensity projections (MIP) of PET/CT images at 72 hours post-injection of 100 μCi (50 μg) ^89^Zr-Df-Bz-NCS-Ab. Signal intensity adjusted to equal scale. All data are presented as mean +/− standard deviation. **B.** Standard uptake values (SUV) obtained from PET/CT image analyses performed at three and seven days post injection. *Day 3: RD-ES = 7 for block (b) and non-block (n/b), LUPI = 3. The same n for day 7.*

**Figure 5:**
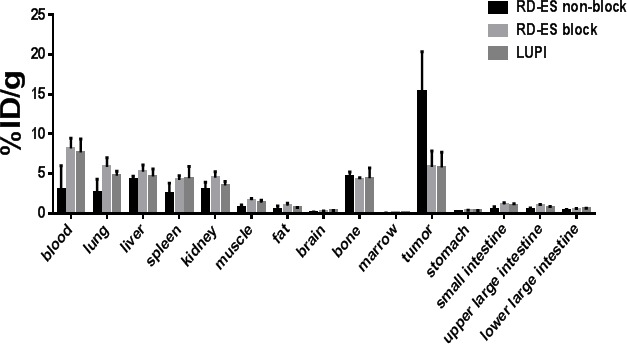
Biodistribution Post PET (Day 7) biodistribution of 89Zr-Df-Bz-NCS-Ab for mice bearing RD-ES (*N* = 4) and LUPI (*N* = 3) xenografts. Data is presented as % ID/g. Blocked animals (*N* = 4) were pre-administered with 1 mg of native antibody to confirm specificity of targeting. All data are presented as mean +/− standard deviation.

One high expressing (RD-ES) and one null expressing (LUPI) cell line were xenografted with 1×10^6^ cells into nude athymic mice and observed for xenograft growth until tumor size reached 100 mm^3^. The mice were then anesthetized with isoflurane and treated with 100 μCi (50 μg) ^89^Zr-labeled Ontuxizumab *via* tail vein injection. PET/CT imaging was performed on post-injection days 3 and 7 and displayed as maximum intensity projections (MIP) of PET/CT images (Figure 4A). ^89^Zr-labeled Ontuxizumab highly and reliably accumulated *in vivo* in RD-ES xenografts, while LUPI xenografts displayed lack of uptake (Figure 4A, middle and right panels, respectively). Background PET activity was not observed. Specificity of the ^89^Zr-labeled Ontuxizumab for the tumor was confirmed by blocking the radiolabeled antibody uptake with cold antibody administration (Figure 4A, left panel). Standard uptake values (SUV) were obtained from PET/CT images at post-injection day 3 and day 7. Significant differences were noted in SUVs between blocked and non-blocked day 3 and day 7 RD-ES xenografts (Figure 4B). The SUV from the day 3 and day 7 PET/CT image analyses of the RD-ES xenografts was significantly higher than that of the non-blocked day 3 and day 7 SUVs for the LUPI xenografts (Figure 4B). *In vivo* biodistribution studies were performed 7 days post-PET/CT imaging; after sacrifice, the organs of interest were harvested, weighed, and radioactivity measured and reported as the percent of injected dose/gram of tissue. This confirmed high radiopharmaceutical uptake in RD-ES non-blocked xenograft relative to normal organs (Figure 5 and Supplemental Table 1). The ratio of tumor to muscle uptake was 21.9 in the RD-ED TEM-1^+^ cell line which was reduced to 3.59 upon blocking, comparable to the TEM-1 low expressing cell line LUPI at 4.3.

**Table 1:** 

Cell Line	Disease	FACS TEM-1Status(−/+/++)	Immunofluorescence(−/+/++)
**LUPI**	Ewing's sarcoma	−	−
**RD-ES**	Ewing's sarcoma	++	++
**SK-ES**	Ewing's sarcoma	+	+
**SYO-1**	Synovial sarcoma	+	+
**FUJI**	Synovial sarcoma	++	++
**ST-88-14**	MPNST	+	+
**SK-LMS-1**	Leiomyosarcoma	−	−
**SK-UT-1**	Uterine sarcoma	−	−
**U2-0S**	Osteosarcoma	−	−
**MG-63**	Osteosarcoma	−	−
**MNNG/HOS**	Osteosarcoma	−	−
**HT-1080**	Fibrosarcoma	−	−
**RD**	Rhabdomyosarcoma	−	−

## DISCUSSION

Sarcomas are an extremely heterogeneous group of tumors comprising over 50 subtypes that are associated with distinctive clinical profiles, response to individual therapies, and prognosis [30]. The reliable identification of TEM-1 in sarcoma may aid in the selection of patients for clinical trials that have the highest potential to benefit from such a targeted therapeutic strategy [5, 6, 19]. However, at this time, the ability to determine the clinically relevant expression of TEM-1 in patients with metastatic disease relies on biopsies of single lesions of primary tumors which may not represent the overall TEM-1 expression in a patient. The use of immunoPET in the imaging of malignancies has increased in use recently, benefiting from advances in PET/CT technology, radionuclide chemistry, and use of improved chelates for radiometal chemistry [4, 5, 7, 25, 31, 32]

We have used FACS analysis and immunofluorescence to identify high and null TEM-1 expressing sarcoma cell lines, and have identified two high expressing cell lines. RD-ES, a Ewing's sarcoma line, and FUJI, a synovial sarcoma line, showed strong binding of the humanized TEM-1 antibody, Ontuxizumab, via FACS analysis and immunofluorescence, with confirmation via Western blotting. Paired cell lines, LUPI (Ewing's sarcoma) and SYO-1 (synovial sarcoma), were identified as null and low expressers of TEM-1, respectively, as were the remainder of the 13 sarcoma cell lines screened in this fashion. Ontuxizumab was successfully conjugated to DFO and labeled with ^89^Zr without altering the antibody's immunoreactivity. It displayed strong affinity to both high-expressing cell lines, RD-ES and FUJI *in vitro*. The *in vivo* utility of ^89^Zr-Ontuxizumab was demonstrated in RD-ES and LUPI xenografts with RD-ES xenografts displaying significantly higher SUVs at post-injection PET imaging at both 3 and 7 days when compared to LUPI xenografts. Blocking of the radiolabeled antibody at both 3 and 7 days post-injection with unconjugated Ontuxizumab demonstrated high specificity of the radiolabeled antibody for the TEM-1 expressing RD-ES xenografts. Biodistribution studies performed 7 days post-injection of the ^89^Zr-Ontuxizumab showed accumulation of the antibody within the target tumor, while non-target tissues and organs of interest showed very little uptake of the antibody.

The sequential process of screening cell lines for a specific monoclonal antibody with subsequent *in vivo* demonstration of ^89^Zr-labeled monoclonal antibody proves the utility of this *in vivo* TEM-1 expression PET test for human clinical screening applications. The findings in this paper await formal clinical trial testing in humans to demonstrate the utility of using radiolabeled Ontuxizumab as the target of the antibody, endosialin, is also expressed on blood vessels. In addition, the pharmacokinetics of the antibody's clearance may be very different in humans than mice, but this awaits formal testing in humans. Clinically, reliable PET imaging of anti-TEM-1 antibody in patients with sarcoma may allow for stratification of patients into cohorts that will attain the greatest benefit from anti-TEM-1 therapy.

## MATERIALS AND METHODS

### Reagents

All experiments involving the use of radioactive materials at Washington University are conducted under the authorization of the Radiation Safety Committee in accordance with the University's Nuclear Regulatory Commission license. Activity was determined with a Capintec CRC-25 Dose Calibrator calibrated to a factor of 465 for ^89^Zr (Capintec, Ramsey, NJ). All chemicals were purchased from Sigma-Aldrich (St. Louis, MO), unless otherwise specified, and all solutions were prepared using ultrapure water with an 18 MΩ-cm resistivity produced by a Millipore Integral 5 water purification system (Millipore, Billerica, MA).

### Cell lines and antibodies

Cell lines were purchased from ATCC, unless otherwise specified. SYO-1 and FUJI were kindly provided by Dr. Akira Kawai (National Cancer Centre Hospital, Tokyo, Japan) and Dr. Kazuo Nagashima (Hokkaido University School of Medicine, Sapporo, Japan) and LUPI was a gift from Dr. John Pfeiffer (Washington University in St. Louis). All complete media was supplemented with 10% FBS and Penicillin-Streptomycin 100x (10,000 U/mL) (Life Technologies, Grand Island, NY). LUPI, RD-ES, SK-ES, FUJI and RD lines were cultured in RPMI Medium 1640 (Life Technologies, Grand Island, NY), SYO-1, ST-88 were maintained in Dulbecco's Modified Eagle Medium (DMEM) (Life Technologies, Grand Island, NY), SK-LMS-1, SK-UT-1, MG63, MNNG/HOS, HT-1080 were cultured in Modified Eagle's Medium (MEM) (Life Technologies, Grand Island, NY), and U2-OS was maintained in McCoy's Medium (Life Technologies, Grand Island, NY). All cell lines other than LUPI, SYO-1 and FUJI are from ATCC and cultured less than six months. The SYO-1 and FUJI cell lines were authenticated by confirming the expression of the pathognomic SYT-SSX fusion gene by RT-PCR and LUPO was authenticated by confirming the expression of the pathognomic EWS-Fli1 fusion gene by RT-PCR. All cell lines were determined to be mycoplasma free using the LookOut Mycoplasma PCR Detection kit. (Sigma-Aldrich, St. Louis, MO).

For cell plating involved in fluorescence-activated cell sorting (FACS) analysis, cells were passaged and plated using 2.5mL of Enzyme-Free Cell Dissociation Buffer (EFCD) (Life Technologies Cat# 13150-016). Anti-TEM-1 antibody (MORAb-004; Ontuxizumab) was provided by Morphotek, Inc. at a stock concentration of 5mg/mL and stored at 4°C. Anti-TEM-1 antibody (1D2.H12.E7.G9) was also provided by Morphotek. Purified TEM-1 antigen was provided by Morphotek, Inc. and stored at −20°C. HRP-Goat anti-Rat IgG + IgM was from Jackson ImmunoResearch (West Grove, PA Cat# 112-035-068), and anti-human beta-actin was from Sigma Aldrich (Cat# A1978). Anti-human IgG-FITC was obtained from Santa Cruz Biotechnology (Santa Cruz, CA, Cat # sc-2456).

### Fluorescence-activated cell sorting and analysis

For the evaluation of TEM-1 expression by FACS, cell lines were plated in a 6-well format at 4×10^5^ cells per well in triplicate. At 24 hours, media was removed from each well and collected into a labeled flow cytometry tube. Each well was washed with 1X phosphate buffered saline (PBS) (Life Technologies, Grand Island, NY) and washings collected. Cells were treated with EFCD for 5-7 minutes (min) at 37°C. Cells were resuspended in PBS and collected and centrifuged at 400g for 5 min and then resuspended in a stock solution of 2% FBS and 2mM EDTA in PBS (stock solution A). Primary labeling with a 1:100 dilution of Ontuxizumab at 4°C for 30 min was performed. After incubation, samples were centrifuged at 400g for 5 min, resuspended and washed with 1X PBS. Secondary labeling was performed with 1:50 dilution of FITC-labeled goat anti-human secondary antibody for 20-30 min at 4°C. After incubation, all samples were centrifuged at 400g for 5 min and washed with PBS. Finally, cell pellets were resuspended in 1%BSA + 2mM EDTA + 1% sodium azide in 1X PBS. The antibody-free unstained sample was provided for each cell line to allow for proper gating and samples without primary antibody labeling were also used to determine background. The cell line (RD-ES) was used as a positive control in all experiments. FACS analysis was performed with a Becton Dickinson FACScan (BD Instruments, San Jose, CA) and FlowJo X (FlowJo, LLC, Ashland, OR).

### Western blot analysis

Four cell lines (RD-ES, LUPI, SYO-1, FUJI) were cultured, passaged, and dissociated utilizing media and agents described above. Cell were collected, placed on ice, and lysed with RIPA buffer over 40 min. Samples were then centrifuged for 15 min at 4°C. Lysates were transferred into a fresh tube with 4X LDS Loading Buffer (Life Technologies). Samples were boiled at 100oC for 5 min, placed on ice, loaded on a NuPAGE 4-12% Bis-Tris Gel 1.0 and run for 55 min at 170 Volts. Protein was transferred onto filters (Novex iBlot 2 PVDF Mini Stacks) via Novex iBlot2 Device (Life Technologies, Grand Island, New York) and blocked with 5% milk in 1x 5% PBS-Tween for 2 hours at room temperature (RT). The primary antibody to TEM-1 (Rat-Anti human 16D2.H12.E7.G9) at a concentration of 1μg/mL was added and incubated overnight at 4°C. Filters were washed three times in 1X PBS-Tween at RT for 15 min 5% PBS-Tween was added to each blot along with HRP-Goat Anti-Rat IgG at a concentration of 1:10,000 and incubated for 1 hour at RT. Filters were washed 3 times in 1X PBS-Tween for 15 min at RT. SuperSignal WestFemto Maximum Sensitivity Substrate Kit (ThermoScientific, Waltham, MA) was used to prepare Femto Streptavidin 1:1 solution and applied to filters. Images were captured *via* Omega Ultra-Lum (Ultralum Inc.; Part #: 910-1024-12; Model: Omega 12iC; Serial#: 6502) and analyzed for TEM-1 expression.

### Semi-quantitative immunofluorescence

All cell lines were plated at 5×10^4^ cells/well onto an 8-chamber slide. At two days after plating, the media was removed from the wells and the slides were incubated in a 4% paraformaldehyde for 10 min at RT. Slides were washed with 1X PBS three times, then incubated in a 1X PBS and 50% goat serum for 30 min at 37°C. Slides were incubated for one hour at 37°C in a 1:100 dilution of primary antibody (Ontuxizumab) diluted in 1X PBS and 50% goat serum. Slides were washed in 1X PBS 3 times and then incubated in a 1:100 dilution of FITC-labeled goat anti-human secondary antibody (sc-2456, Santa Cruz Biotech) for 1 hour at 37°C. Slides were washed in 1X PBS 3 times, and mounted in Dapi/Antifade solution (SlowFade Gold antifade reagent with DAPI). Images were taken with a locked exposure time of 25 milliseconds using a 60x objective for FITC to allow for semi-quantitative microscopy across all images.

### Animal models

Nude athymic mice (Nu/J homozygous, Cat#002019, The Jackson Lab) were xenografted with 1×10^6^ RD-ES or LUPI cells that had been resuspended in 1X PBS 30% Matrigel (Corning, Inc., Cat # CB-40234). Mice were anesthetized with 1-2% isofluorane and injected at the left shoulder for RD-ES xenografts and injected at the right shoulder for LUPI xenografts. Mice were observed until tumor size was 100mm^3^ and then underwent small animal PET/CT imaging.

### ^89^Zr production and purification

^89^Zr was produced via the ^89^Y(p,n)^89^Zr reaction using the CS-15 cyclotron (Cyclotron Corporation, Berkeley, CA) and separated *via* ion exchange chromatography using an in-house automated system as previously described [22-24], with a resulting specific-activity of 22-134 mCi/μmole.

### Radiolabeling of anti-TEM-1 antibody

^89^Zr labeling was performed according to previously published methods [25, 26]. Briefly, Ontuxizumab (5 mg/ml) was conjugated to isothiocyanatobenzyl-desferrioxamine-NCS (Df-Bz-NCS) (Macrocyclics, Dallas, TX) at 1:10 (Antibody) Ab to chelate ratio for 1 hour at 37°C in the presence of 0.1 M sodium carbonate buffer (pH 9). The unreacted chelate was removed utilizing Zeba desalting columns (40,000 MW cut off, 0.5 mL volume, ThermoFisher Scientific, Rockford, IL). The Ab-Df-Bz-NCS conjugate was combined with ^89^Zr oxalate at 1:1 or 1:5 mg/mCi and pH 7.1. The mixture was incubated for one hour at 37°C. The labeling efficiency was confirmed by radio-iTLC on glass microfiber chromatography paper impregnated with silica gel (Agilent Technologies, Lake Forest, CA) with 50 mM diethylene triamine pentaacetic acid (DTPA) eluent solvent and a Bioscan AR- 2000 radio-TLC scanner equipped with a 10% methane/argon gas supply. The specific activity obtained was 1-5 μCi/μg with labeling efficiency of 98-100%; therefore, the compounds were used with no further purification.

### *In vitro* cell binding

Cells were grown to ∼50% confluence in 6-well tissue culture plates and incubated with 1-2 μCi (0.5 μg) of radiolabeled Ontuxizumab in 500 μL of complete media for 35 min at 37°C. Unbound antibody was removed by washing cells thrice with PBS. Cells were then solubilized in PBS containing 0.1% SDS, the lysate was transferred to microfuge tubes and cell associated activity detected with a gamma-counter. Extra wells with untreated cells corresponding to each cell line were assessed for cell number and used as reference. The results were expressed as counts per min per 5 x10^5^ cells (CPM/5×10^5^ cells).

### Internalization assay

Cells were plated in 24-well tissue culture plates at 1×10^5^ cells/well and incubated with 1-2 μCi (0.5 μg) of radiolabeled antibody in 250 μl of complete media per well for 0.5, 1, 2, 4, 6, and 24 hours in humidified atmosphere supplemented with 5% CO_2_ at 37°C. Unbound antibody was removed by washing cells three times with PBS. Cells were then harvested by incubating in 250 μl of 0.05% Trypsin-EDTA (Thermo Fisher) for 5 minutes at 37°C. Cells were pelleted, the supernatant collected, and the pellet re-suspended in 250 μl of 20 mM sodium citrate/150 mM sodium chloride, pH 2.5 for 2 minutes at 22°C to remove the membrane associated antibody. Cells were pelleted, the supernatant collected and combined with the Trypsin supernatant to represent the membrane bound antibody. The cell pellet was washed once with PBS. The activity associated with the internalized (cell pellet) and membrane bound (supernatants) was determined with a gamma-counter.

### *In vivo* Biodistribution

Biodistribution studies were conducted immediately after 7 days post injection PET/CT imaging studies. Briefly, 100 μCi (50 ug) ^89^Zr-Df-Bz-NCS-Ab were tail vein injected. Animals were sacrificed, organs of interest were harvested, weighed, and associated radioactivity determined on a γ-counter. After correcting for background and decay, the percent-injected dose per gram (%ID/gram) and percent-injected dose per organ (%ID/organ) were calculated by comparison to a weighed, counted standard.

### Small animal PET/CT imaging

Prior to imaging, mice were injected intravenously (tail vein) with 100 μCi of ^89^Zr-Df-Bz-NCS-Ab. At three and seven days post-injection mice were anaesthetized with 1%-2% isoflurane and imaged with an Inveon small animal PET/CT scanner (Siemens Medical Solutions). Static images were collected for 20 min and reconstructed with the Maximum A Posteriory (MAP) probability algorithm [27] followed by co-registration with the Inveon Research Workplace 4.0 (IRW) image display software (Siemens Medical Solutions, Knoxville, TN). Regions of interest (ROI) were selected from PET images using CT anatomical guidelines and the activity determined with IRW software. Standard uptake values (SUV) were determined as nCi/cc x animal weight/injected dose. To block the binding of ^89^Zr labeled antibody, 40 mg/kg of unlabeled antibody was injected intravenously (tail vein) 10 minutes prior to the labeled antibody.

### Statistical analysis

Data are expressed as mean ± standard deviation (SD) unless noted otherwise. Statistical significance was determined using the unpaired, 2-tailed Student *t*-test and 95% confidence level. P values less than 0.05 were considered significant. All data were analyzed and plotted using GraphPad Prizm version 5.0 or MEDCalc.

## SUPPLEMENTARY MATERIAL FIGURES AND TABLE



## References

[1] Buckanovich RJ, Sasaroli D, O'Brien-Jenkins A, Botbyl J, Hammond R, Katsaros D, Sandaltzopoulos R, Liotta LA, Gimotty PA, Coukos G (2007). Tumor vascular proteins as biomarkers in ovarian cancer. Journal of clinical oncology.

[2] Rmali KA, Puntis MC, Jiang WG (2005). Prognostic values of tumor endothelial markers in patients with colorectal cancer. World journal of gastroenterology : WJG.

[3] Liu S, Li D, Park R, Liu R, Xia Z, Guo J, Krasnoperov V, Gill PS, Li Z, Shan H, Conti PS (2013). PET imaging of colorectal and breast cancer by targeting EphB4 receptor with 64Cu-labeled hAb47 and hAb131 antibodies. Journal of nuclear medicine.

[4] Marquez BV, Ikotun OF, Zheleznyak A, Wright B, Hari-Raj A, Pierce RA, Lapi SE (2014). Evaluation of (89)Zr-pertuzumab in Breast cancer xenografts. Molecular pharmaceutics.

[5] Chacko AM, Li C, Nayak M, Mikitsh JL, Hu J, Hou C, Grasso L, Nicolaides NC, Muzykantov VR, Divgi CR, Coukos G (2014). Development of 124I immuno-PET targeting tumor vascular TEM1/endosialin. Journal of nuclear medicine.

[6] Bagley RG (2009). Endosialin: from vascular target to biomarker for human sarcomas. Biomarkers in medicine.

[7] Paudyal B, Paudyal P, Oriuchi N, Hanaoka H, Tominaga H, Endo K (2011). Positron emission tomography imaging and biodistribution of vascular endothelial growth factor with 64Cu-labeled bevacizumab in colorectal cancer xenografts. Cancer science.

[8] Teicher BA (2007). Newer vascular targets: endosialin (review). International journal of oncology.

[9] Carson-Walter EB, Watkins DN, Nanda A, Vogelstein B, Kinzler KW, St Croix B (2001). Cell surface tumor endothelial markers are conserved in mice and humans. Cancer research.

[10] Christian S, Ahorn H, Koehler A, Eisenhaber F, Rodi HP, Garin-Chesa P, Park JE, Rettig WJ, Lenter MC (2001). Molecular cloning and characterization of endosialin, a C-type lectin-like cell surface receptor of tumor endothelium. The Journal of biological chemistry.

[11] St Croix B, Rago C, Velculescu V, Traverso G, Romans KE, Montgomery E, Lal A, Riggins GJ, Lengauer C, Vogelstein B, Kinzler KW (2000). Genes expressed in human tumor endothelium. Science.

[12] Tomkowicz B, Rybinski K, Foley B, Ebel W, Kline B, Routhier E, Sass P, Nicolaides NC, Grasso L, Zhou Y (2007). Interaction of endosialin/TEM1 with extracellular matrix proteins mediates cell adhesion and migration. Proceedings of the National Academy of Sciences of the United States of America.

[13] Becker R, Lenter MC, Vollkommer T, Boos AM, Pfaff D, Augustin HG, Christian S (2008). Tumor stroma marker endosialin (Tem1) is a binding partner of metastasis-related protein Mac-2 BP/90K. FASEB journal.

[14] Rettig WJ, Garin-Chesa P, Healey JH, Su SL, Jaffe EA, Old LJ (1992). Identification of endosialin, a cell surface glycoprotein of vascular endothelial cells in human cancer. Proceedings of the National Academy of Sciences of the United States of America.

[15] Christian S, Winkler R, Helfrich I, Boos AM, Besemfelder E, Schadendorf D, Augustin HG (2008). Endosialin (Tem1) is a marker of tumor-associated myofibroblasts and tumor vessel-associated mural cells. The American journal of pathology.

[16] Zhao A, Nunez-Cruz S, Li C, Coukos G, Siegel DL, Scholler N (2011). Rapid isolation of high-affinity human antibodies against the tumor vascular marker Endosialin/TEM1, using a paired yeast-display/secretory scFv library platform. Journal of immunological methods.

[17] Rouleau C, Curiel M, Weber W, Smale R, Kurtzberg L, Mascarello J, Berger C, Wallar G, Bagley R, Honma N, Hasegawa K, Ishida I, Kataoka S, Thurberg BL, Mehraein K, Horten B (2008). Endosialin protein expression and therapeutic target potential in human solid tumors: sarcoma versus carcinoma. Clinical cancer research.

[18] Davies G, Cunnick GH, Mansel RE, Mason MD, Jiang WG (2004). Levels of expression of endothelial markers specific to tumour-associated endothelial cells and their correlation with prognosis in patients with breast cancer. Clinical & experimental metastasis.

[19] Rouleau C, Smale R, Fu YS, Hui G, Wang F, Hutto E, Fogle R, Jones CM, Krumbholz R, Roth S, Curiel M, Ren Y, Bagley RG, Wallar G, Miller G, Schmid S (2011). Endosialin is expressed in high grade and advanced sarcomas: evidence from clinical specimens and preclinical modeling. International journal of oncology.

[20] Rouleau C, Sancho J, Campos-Rivera J, Teicher BA (2012). Endosialin expression in side populations in human sarcoma cell lines. Oncology letters.

[21] Stillebroer AB, Franssen GM, Mulders PF, Oyen WJ, van Dongen GA, Laverman P, Oosterwijk E, Boerman OC (2013). ImmunoPET imaging of renal cell carcinoma with (124)I- and (89)Zr-labeled anti-CAIX monoclonal antibody cG250 in mice. Cancer biotherapy & radiopharmaceuticals.

[22] Verel I, Visser GW, Boellaard R, Stigter-van Walsum M, Snow GB, van Dongen GA (2003). 89Zr immuno-PET: comprehensive procedures for the production of 89Zr-labeled monoclonal antibodies. Journal of nuclear medicine.

[23] Holland JP, Sheh Y, Lewis JS (2009). Standardized methods for the production of high specific-activity zirconium-89. Nuclear medicine and biology.

[24] Wooten AL, Madrid E, Schweitzer GD, Lawrence LA, Mebrahtu E, Lewis BC, Lapi SE (2013). Routine Production of Zr-89 Using an Automated Module. Appl Sci-Basel.

[25] Zheleznyak A, Ikotun OF, Dimitry J, Frazier WA, Lapi SE (2013). Imaging of CD47 expression in xenograft and allograft tumor models. Molecular imaging.

[26] Holland JP, Caldas-Lopes E, Divilov V, Longo VA, Taldone T, Zatorska D, Chiosis G, Lewis JS (2010). Measuring the pharmacodynamic effects of a novel Hsp90 inhibitor on HER2/neu expression in mice using Zr-DFO-trastuzumab. PloS one.

[27] Qi J, Leahy RM (2000). Resolution and noise properties of MAP reconstruction for fully 3-D PET. IEEE transactions on medical imaging.

[28] Rybinski K, Imtiyaz HZ, Mittica B, Drozdowski B, Fulmer J, Furuuchi K, Fernando S, Henry M, Chao Q, Kline B, Albone E, Wustner J, Lin J, Nicolaides NC, Grasso L, Zhou Y (2015). Targeting endosialin/CD248 through antibody-mediated internalization results in impaired pericyte maturation and dysfunctional tumor microvasculature. Oncotarget.

[29] Rouleau C, Gianolio DA, Smale R, Roth SD, Krumbholz R, Harper J, Munroe KJ, Green TL, Horten BC, Schmid SM, Teicher BA (2015). Anti-Endosialin Antibody-Drug Conjugate: Potential in Sarcoma and Other Malignancies. Mol Cancer Ther.

[30] Fletcher CDM BJ, Hogendoorn PCW, Mertens F (2013). World Health Organization Classification of tumours of soft tissue and bone.

[31] Dijkers EC, Oude Munnink TH, Kosterink JG, Brouwers AH, Jager PL, de Jong JR, van Dongen GA, Schroder CP, Lub-de Hooge MN, de Vries EG (2010). Biodistribution of 89Zr-trastuzumab and PET imaging of HER2-positive lesions in patients with metastatic breast cancer. Clinical pharmacology and therapeutics.

[32] Gaykema SB, Brouwers AH, Lub-de Hooge MN, Pleijhuis RG, Timmer-Bosscha H, Pot L, van Dam GM, van der Meulen SB, de Jong JR, Bart J, de Vries J, Jansen L, de Vries EG, Schroder CP (2013). 89Zr-bevacizumab PET imaging in primary breast cancer. Journal of nuclear medicine.

