# Supplementary material for: Development of 89Zr-Ontuxizumab for in vivo TEM-1/endosialin PET applications
Source: Oncotarget. 2016 Feb 21;7(11):13082–92. doi: 10.18632/oncotarget.7552 (PMC4914343; doi:10.18632/oncotarget.7552)
Supplement: Supplementary file 1 [file oncotarget-07-13082-s001.pdf]

Development of ^{89}Zr-Ontuxizumab for *in vivo* TEM-1/endosialin PET applications

Supplementary Material

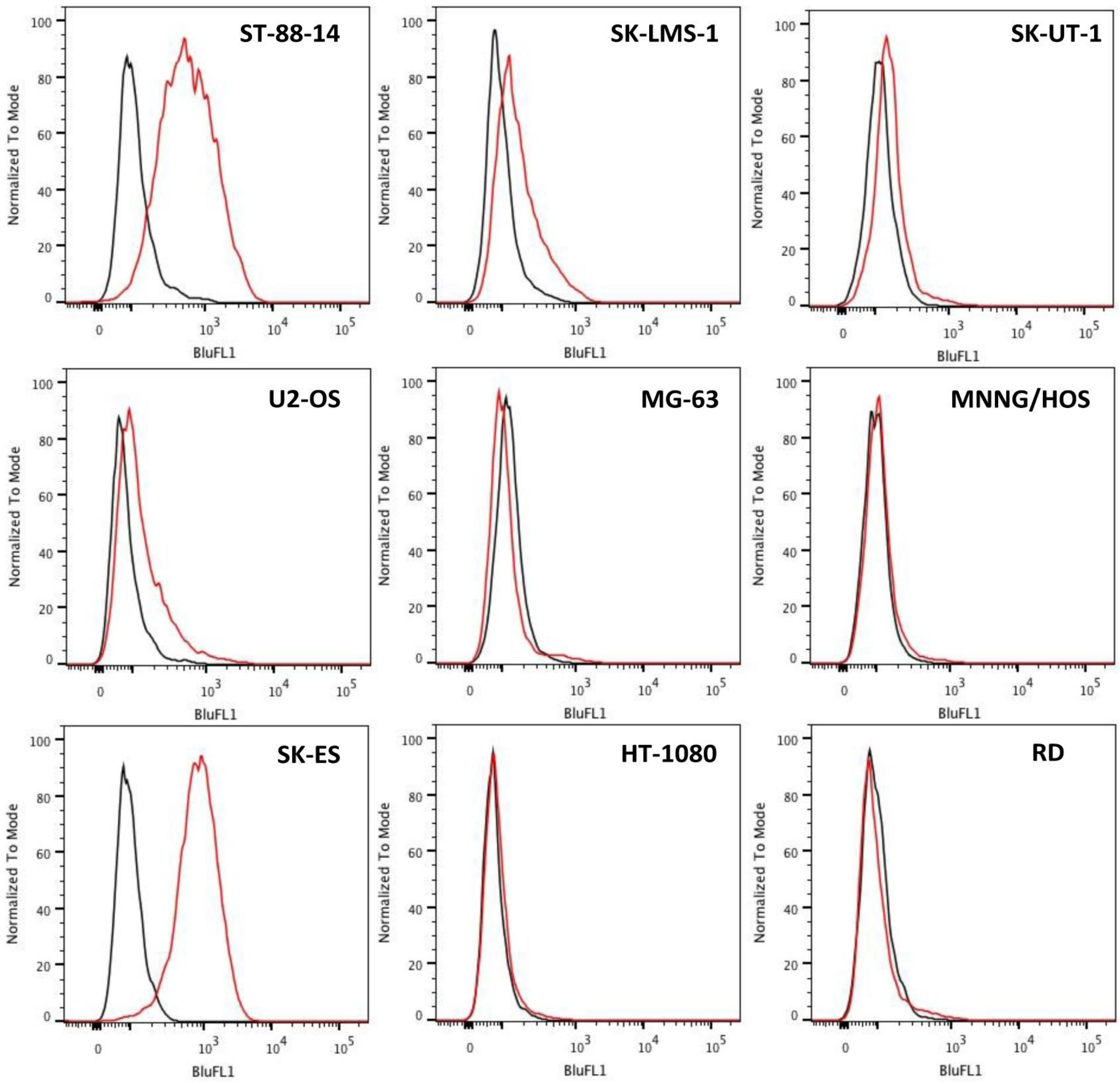

Supplemental Figure 1: FACS Analysis of TEM-1 Expression in Additional Sarcoma Cell lines. FACS analysis of anti-TEM-1 antibody expression of the nine other sarcoma cell lines. With the exceptions of SK-ES and ST-88-14 displaying intermediate expression of TEM-1, the remainder of sarcoma cells lines is negative for TEM-1 expression.

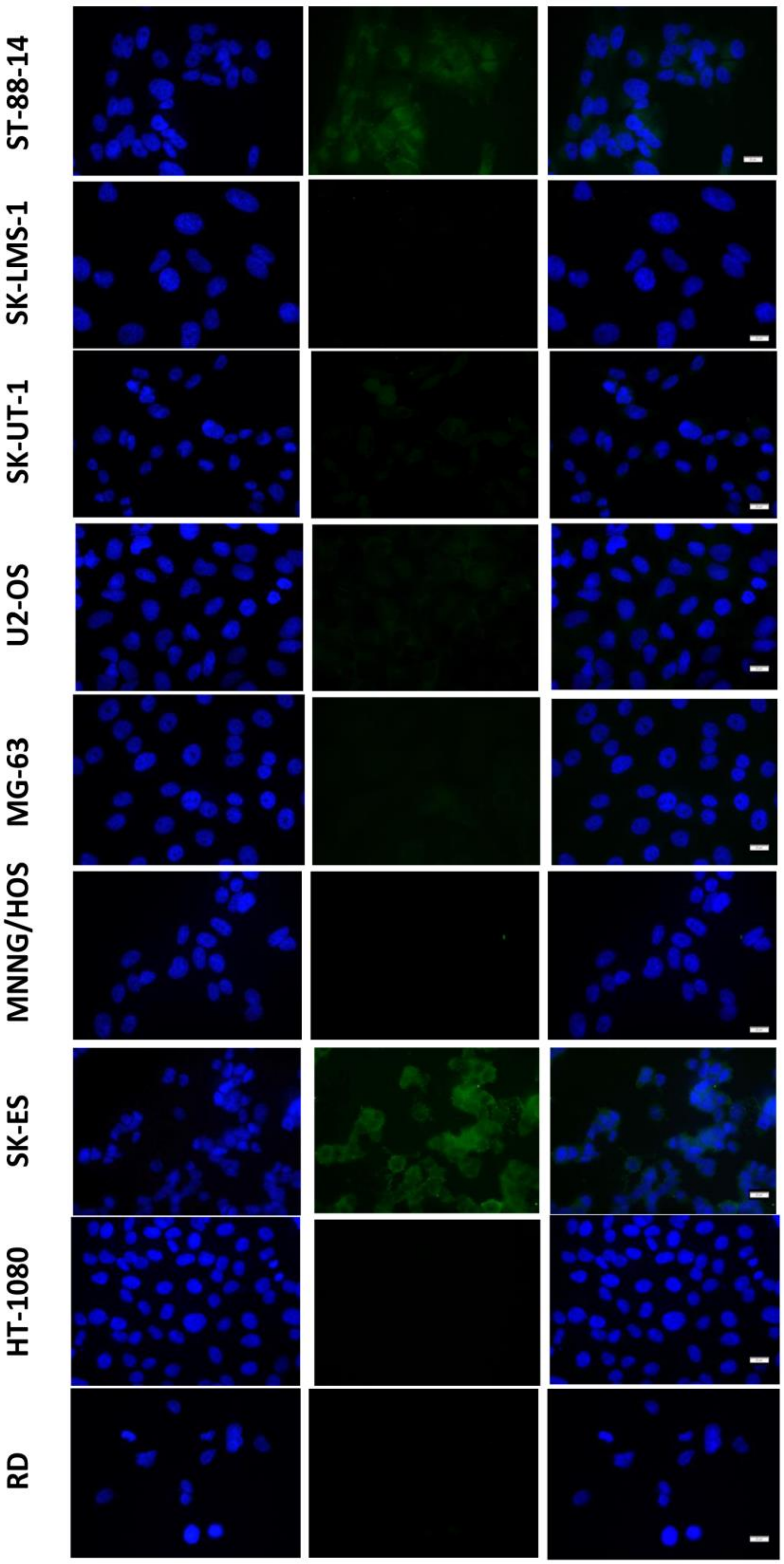

Supplemental Figure 2: Immunofluorescence for TEM-1 Expression of Additional Sarcoma Cell Lines. With the exceptions of SK-ES and ST-88-14 displaying intermediate expression of TEM-1, the remainder of sarcoma cells lines only background level of expression as seen by IF. This is in agreement with Supplemental FIGURE 1 FACS expression.

Supplementary Table 1:

Tissue Type	RD-ES non-block (%ID/g)		RD-ES block (%ID/g)		LUPI (%ID/g)	
	Mean	Std Dev	Mean	Std Dev	Mean	Std Dev
blood	2.93	3.06	8.16	1.31	7.66	1.71
lung	2.56	1.74	5.82	1.17	4.72	0.59
liver(all)	4.22	0.44	5.25	0.83	4.63	0.95
spleen	2.46	1.34	4.21	0.53	4.43	1.49
kidney	2.92	0.97	4.47	0.77	3.49	0.53
muscle	0.70	0.34	1.65	0.21	1.34	0.32
fat	0.52	0.40	0.95	0.32	0.68	0.05
brain	0.09	0.08	0.25	0.03	0.29	0.09
bone	4.65	0.57	4.30	0.19	4.40	1.32
marrow	0.02	0.01	0.03	0.01	0.03	0.01
tumor	15.33	5.03	5.84	2.01	5.78	1.93
stomach	0.16	0.06	0.31	0.07	0.29	0.08
small intestine	0.49	0.34	1.09	0.25	1.01	0.18
upper large intestine	0.41	0.27	0.91	0.20	0.75	0.11
lower large intestine	0.32	0.15	0.52	0.10	0.56	0.07
